# Is childbirth location associated with higher rates of favourable early breastfeeding practices in Sub-Saharan Africa?

**DOI:** 10.7189/jogh.09.010417

**Published:** 2019-06

**Authors:** Natasha Bergamaschi, Laura Oakley, Lenka Benova

**Affiliations:** 1 Faculty of Epidemiology and Population Health, London School of Hygiene and Tropical Medicine, London, UK; 2 Ealing Hospital, London North West University Healthcare NHS Trust, London, United Kingdom; 3 Centre for Fertility and Health, Norwegian Institute of Public Health, Oslo, Norway; 4 Department of Public Health, Institute of Tropical Medicine, Antwerp, Belgium

## Abstract

**Background:**

Favourable early breastfeeding practices have a beneficial impact throughout an infants’ lifespan. Childbirth location is likely to affect these practices through support during the intrapartum and immediate postpartum period. This study aimed to investigate the association between childbirth location and favourable early breastfeeding practices in Sub-Saharan Africa (SSA).

**Methods:**

Demographic and Health Survey (2000–2013) data across 30 SSA countries were utilised. Childbirth location was categorised as home vs facility, and further into public vs private sector. Early breastfeeding practices included: early initiation of breastfeeding (EIBF) (within 1 hour of birth), and no prelacteal feeding (fed only breast milk in the first 3 days). Multivariate logistic regression models adjusted for confounders were used to assess this association.

**Results:**

Overall, 50.0% (country range 32.6%-95.5%) of infants received EIBF and 61.0% had no prelacteal feeding. Compared with home births, facility deliveries had higher adjusted odds of EIBF (adjusted odds ratio, aOR = 1.39, 95% confidence interval (CI) = 1.30-1.48, *P* < 0.001) and no prelacteal feeding (aOR = 1.75, 95% CI = 1.63-1.89, *P* < 0.001). Private sector facilities had lower adjusted odds of no prelacteal feeding (aOR = 0.89, 95% CI = 0.81-0.99, *P* = 0.036) when compared to public sector facilities. There was no evidence to suggest delivery sector was associated with EIBF (aOR = 0.93, 95% CI = 0.85-1.03, *P* = 0.212).

**Conclusions:**

This study showed early breastfeeding practices are suboptimal and are associated with delivery location in SSA. Further research is required to better understand how characteristics of care may explain these patterns in order to improve feeding practices.

The benefits of breastfeeding have an influence throughout the entire lifespan for the infant [1], and also provide many benefits for the mother – including birth spacing and reducing her risk of diabetes, breast and ovarian cancers [2,3]. Alongside reduced infant mortality, breastfed infants also have improved nutritional status and lower risk of respiratory and diarrhoeal diseases – two major causes of infant mortality, especially in Sub-Saharan Africa (SSA) [4-6]. In later life, having been breastfed is protective against chronic diseases, including inflammatory bowel disease and diabetes [6]. Furthermore, optimal breastfeeding practices are paramount to achieving several of the Sustainable Development Goals (SDGs), including SDGs 2, 3 and 4 [7]. Better maternal and child health, reduced health care costs, increased cognitive development and an enhanced workforce all contribute to wider society, with positive impacts on a country’s economy [8].

Current WHO and UNICEF guidelines recommend that breastfeeding is initiated within one hour of birth and continued with no other foods/liquids for the first 6 months (exclusive breastfeeding). They also recommend breastfeeding be continued until at least 24 months of age alongside other age-appropriate foods [9]. Delayed initiation of breastfeeding (>1 hour following birth) is correlated with a higher incidence of prelacteal feeding (any food/liquid other than breast milk given in the first 3 days of life), the discarding of colostrum (first nutrient and immune-dense breast-milk) and an increased risk of neonatal mortality [10-14]. No prelacteal feeding, defined as only breast milk given to the infant in the first 3 days of life, has been found to prevent later detrimental breastfeeding practices [15,16]; all prelacteal substances are less nutritious and beneficial for the infant. Contaminants may be introduced through the food/liquid and likely hinder the establishment of breastfeeding [17].

The importance of childbirth location to early breastfeeding practices is through support and enablement around the intrapartum and immediate postpartum period. Previous research has looked at childbirth location as a determinant of early breastfeeding practices using mainly binary categorisation (home vs facility) [18-21]. These studies generally found more favourable breastfeeding outcomes in infants born within facilities, although classifications of health facilities varied across the studies. A descriptive analysis of sector of childbirth facility in 57 LMICs found that early breastfeeding outcomes were more favourable amongst public facility deliveries [22], but we have not identified any cross-country research adjusted for confounders assessing the effect of sector of delivery facility on early breastfeeding practices in SSA.

Recent data (2010-2015) found West and Central Africa overall prevalence of EIBF was lower than the WHO target of 50% by 2025 [23]. The objective of this study was to examine the association between childbirth location (home vs facility, within facility: private vs public sector) and favourable early breastfeeding outcomes (EIBF and no prelacteal feeding) across 30 SSA countries. We focus on SSA because it is the region with the largest number of countries with a recent Demographic and Health Surveys (DHS) and there is substantial benefit to infant survival to be gained by improving early breastfeeding practices.

## METHODS

### Data and population

We used data from the most recent DHS, a nationally-representative household survey collected between 2000 - 2013 for 30 countries in SSA, over 80% of the population in SSA is represented by these surveys, Appendix S1 in **Online Supplementary Document** [24]. The combined data set provided 103 611 observations relating to the most recent live birth (last child reported in case of multiples) in the two years preceding the survey for women aged 15-49 at the time of survey. Data collected on births and breast-feeding practices were self-reported by women.

Observations were excluded if data on delivery location was missing or unclassified, if the child died within 1-month of birth or, if they were no longer alive at the time of the survey. Infants who died within 1-month of birth were excluded on the basis that they might have been less likely to be breastfed due to poor health or prematurity. Facility deliveries are also more likely with these infants, due to complications during pregnancy or during labour that resulted in presentation to care facilities.

### Definitions

The exposure – childbirth location was described by two variables; delivery location, and delivery sector. A delivery in any home environment was classified as a home birth, whereas a delivery within a public or private sector health facility was classified as a facility birth. For facility deliveries, observations were further divided by ‘delivery sector’ – public or private. Women giving birth in a government hospital, health centre, health post or other public sector facilities were classified as a public-sector facility delivery. Any deliveries within a private hospital/clinic, including for profit and not-for-profit (NGOs and faith-based providers) were considered as private-sector facility births. Deliveries taking place at home were not further differentiated by sector and were excluded from analyses of sector within the subset of facility deliveries.

Both outcomes were derived from the data and previously used in a descriptive analysis. The first outcome; infants put to the breast within one hour of birth, is a WHO recommended indicator for assessing Infant and Young Child Feeding (IYCF) [25]. The second outcome describes infants who only received breast milk in the first three days after birth (no prelacteal feeding).

### Analysis

Initially a univariate logistic regression analysis was conducted, tabulating all variables considered in our conceptual framework (Appendix Figure S1 in **Online Supplementary Document**), both exposures and both outcomes. Variables included maternal age group (5-year age groups), maternal education (no education, primary, secondary and higher), marital status (never married and ever married), household wealth quintile, sex of infant, parity (1, 2-3, 4-5, 6+), multiple births (yes, no), mode of delivery (vaginal birth, c-section), wantedness of child (wanted then, wanted later, wanted no more), number of ANC visits (no visits, 1-3, 4+), and residence (urban and rural). Any variable that showed an association with childbirth location and/or outcome measures was viewed as a potential confounder and considered for inclusion in the final multivariate analysis model. A bivariate analysis, adjusted only for country (fixed-effect), was also conducted.

Multivariable analysis consisted of four logistic regression models (**Table 1**). A priori confounders (maternal age group, wealth quintile and maternal education) were first added, followed by variables in order of proximity to the exposures and outcomes in the conceptual framework. Likelihood ratio tests were used to assess the improvement in model fit related to addition of each variable. All analyses were carried out using Stata 14 (Stata Corp, College Station, TX, USA).

**Table 1:** Description of final logistic regression models

Model	Exposure	Outcome
**Model 1**	Facility delivery (reference: home delivery)	Initiation of breastfeeding within 1 hour
**Model 2**		No prelacteal feeding
**Model 3**	Among facility births only: Private sector delivery (reference: public sector delivery)	Initiation of breastfeeding within 1 hour
**Model 4**		No prelacteal feeding

In order to increase sample size for certain areas and subgroups and thus decrease sample variability, DHS samples are selected with unequal probability of selection. Women in each DHS survey have an individual sample weight that is used to calculate country-level representative summary statistics. In pooled analyses we applied weights that accounted for both country-specific survey design and each country’s population size relative to the combined population of all 30 countries included in analysis, to ensure that estimates are representative of the population residing in study countries.

For variables with >1% missingness, we assessed the association of the missingness with exposure and outcome. All missing data for any variable were excluded from the final analysis using the logistic regression model.

Educational level, marital status, mode of delivery and wantedness of child all had some missing data, the former two had <0.01% missing, whilst both latter variables had <1% missingness. Number of ANC visits had 2.38% missingness, across all countries included in the data set. This was considered significant missingness and therefore was analysed for any association with the exposures and outcomes.

### Ethics

The DHS received institutional review centrally (ICF International), and approval by every participating country. This study received ethical approval from the Research Ethics Committee of the London School of Hygiene and Tropical Medicine, UK.

## RESULTS

Overall in the 30 SSA countries included, 46.9% (95% CI = 45.9-47.9) of women delivered in a facility, of which 77.8% (95% CI = 76.7-78.9) delivered within the public sector (**Table 2**). Around half of women – 50.02% (95% CI = 49.2-50.8) initiated breastfeeding within 1 hour of birth and 61.0% (95% CI = 60.1-61.8) of newborns had only breast milk in the first three days of life (no prelacteal feeding).

**Table 2:** Descriptive analysis of analysis sample n = 103 611, representing 30 sub-Saharan African countries

Variables	Categories	Frequency (n)	Percentage (%)*
Delivery location (N = 103 611)	Home	44 891	53.09
	Facility	58 720	46.91
Delivery sector (Among facility deliveries only), (N = 58 720)	Public	50 174	77.82
	Private	8546	22.18
Early breastfeeding (N = 103 611)	Within 1 h	57 068	50.02
	>1 h	46 543	49.98
No prelacteal feeding (N = 103 611)	Yes	66 801	61.01
	No	36 810	38.99
Maternal age group (N = 103 611)	15-19	11 239	9.78
	20-24	27 007	25.69
	25-29	27 561	27.27
	30-34	18 993	18.62
	35-39	12 388	12.28
	40-44	5039	4.87
	45-49	1384	1.50
Educational level (N = 103 611)	No education	45 210	41.74
	Primary	36 975	36.67
	Secondary and higher	21 423	21.59
	Missing	3	<0.01
Marital status (N = 103 611)	Never married	6459	4.00
	Ever married	97 149	95.99
	Missing	3	<0.01
Household wealth quintile (N = 103 611)	Poorest	24 906	22.76
	Poorer	22 058	22.19
	Middle	20 548	20.05
	Richer	19 160	18.75
	Richest	16 939	16.24
Sex of infant (N = 103 611)	Male	52 138	50.53
	Female	51 473	49.47
Parity(N = 103 611)	1	21 773	19.77
	2-3	35 838	34.04
	4-5	23 539	22.82
	>5	22 461	23.37
Multiple births (N = 103 611)	Yes	101 879	1.49
	No	1732	98.51
Mode of delivery (N = 103, 611)	Vaginal	99 431	96.62
	C-section	3974	3.38
	Missing	206	<1.00
Wantedness of child (N = 103 611)	Wanted then	73 286	72.20
	Wanted later	21 214	19.36
	Wanted no more	8968	8.44
	Missing	143	<1.00
Antenatal care (N = 103 611)	No ANC	15 600	22.34
	1-3 visits	37 883	32.51
	4+ visits	47 659	41.90
	Missing	2469	3.25
Residence (N = 103 611)	Urban	29 076	25.29
	Rural	74 535	74.71

ANC – antenatal care

*Weighted by within country survey weights and country population sizes.

Gabon had the highest percentage of women delivering in a facility at 92.9%, and Ethiopia the lowest – 11.4% (Appendix S1 in **Online Supplementary Document**). Among women who delivered in facilities, public sector deliveries were a large majority, ranging from 57.4% in Nigeria to 98.8% in Rwanda. Malawi had the highest percentage of women initiating breastfeeding within 1 hour – 95.5%; and the lowest was in Chad where only 32.6% of women met the definition of this practice. The cross-country range for no prelacteal feeding was from 2.7% in Chad to 96.8% in Malawi.

Univariate analysis found that maternal age, education, wealth, marital status, residence, parity, number of ANC visits, mode of delivery, wantedness of pregnancy and multiple births were all associated with facility deliveries. With the exception of the latter two variables, all other variables were also associated with delivery sector.

### Home vs facility deliveries

Bivariate associations, adjusted only for country (fixed effect) are displayed in **Table 3**, alongside adjusted odds ratios (full adjusted models are shown in Table S2 in **Online Supplementary Document**). Once adjusted for all other confounders the odds ratio for the association between facility delivery and EIBF was 1.39 (Model 1, 95% CI = 1.30-1.48, *P* < 0.001). Of the 30 countries, this association was also positive and significant (*P* < 0.05) in 19 countries, negative and significant in two countries, and no association was seen in nine countries [Appendix S3 in **Online Supplementary Document**]. Model 2 (bivariate analysis of facility delivery and no prelacteal feeding) found women delivering within facilities had 2.00 times the odds of giving only breast milk within the first three days when compared to women delivering at home (95% CI = 1.88-2.10, *P* < 0.001). Once adjusting for confounders there was a change in OR resulting in an adjusted odds ratio of 1.75 (95% CI = 1.63-1.89, *P* < 0.001). Of the 30 countries, this association was positive and significant (*P* < 0.005) in 19 countries.

**Table 3:** Bivariate and adjusted odds ratios for logistic regression models 1-4

Model	Exposure	Outcome		Bivariate model*	Adjusted model†
1	Facility delivery	Initiation of breastfeeding within 1 hour	**OR**	**1.40**	**1.39**
			*P-*value	<0.001	<0.001
			95% CI	1.32-1.48	1.30-1.48
			n	103 611	100 799
2		No prelacteal feeding	**OR**	**2.00**	**1.75**
			*P-*value	<0.001	<0.001
			95% CI	1.88-2.1	1.63-1.89
			n	103 611	100 799
3	Private sector	Initiation of breastfeeding within 1 hour	**OR**	**0.91**	**0.93**
			*P-*value	0.062	0.212
			95% CI	0.82-1.00	0.85-1.03
			n	58 720	57 051
4		No prelacteal feeding	**OR**	**0.88**	**0.89**
			*P-*value	0.015	0.036
			95% CI	0.79-0.97	0.81-0.99
			n	58 720	57 051

OR – odds ration, P – value of Wald test

*Adjusted for country only.

†Adjusted for maternal age group, parity, maternal education, mode of delivery (model 3 and 4 only), number of ANC visits, sex of infant, multiple births, wantedness of child, wealth quintile, residence, marital category and country.

### Public vs private sector deliveries

EIBF was higher amongst women delivering within the public sector - 56.9% vs 50.1% in the private sector. No prelacteal feeding was also higher for deliveries taking place in the public sector – 7 2.9% vs 67.4% in the private sector. Model 3 looking at the bivariate analysis of delivery sector and EIBF did not find a significant association, after adjusting for confounders there continued to be no evidence of a significant association between delivery sector deliveries and EIBF (OR = 0.93, 95% CI = 0.85-1.03, *P* = 0.212). Across the 30 countries, there was no significant association (*P*<0.05) in 23 countries, three countries had a positive significant, three a negative significant association between EIBF and private facility delivery, and one country did not have a sufficiently large sample size of deliveries in the private sector to examine this association. Model 4 (bivariate analysis of private sector delivery and no prelacteal feeding) found women delivering in private facilities had 0.88 times the odds of no prelacteal feeding when compared to women delivering in the public sector (95% CI = 0.79-0.97, *P* = 0.015). After adjusting for confounders this changed marginally to OR = 0.89 (95% CI = 0.81-0.99, *P* = 0.036). Of the 30 countries, this association was negative and significant in five countries, not significant in 24 and one country showed a positive significant association.

## DISCUSSION

To our knowledge, this is the first analysis, comprising of recent DHS data looking at the independent association between childbirth location and favourable early breastfeeding practices across multiple countries in SSA. Specifically, the inclusion of delivery sector as an explanatory variable is a unique feature of this analysis.

We found that EIBF and no prelacteal feeding vary widely across the 30 SSA countries included. Half of mothers in SSA initiate breastfeeding within one hour of birth and 61% of newborns receive only breast-milk in the first three days post-partum; on the whole this reflects suboptimal practices and is in need of immediate improvement to achieve better health of children and mothers. Our study provides crucial evidence that the odds of achieving these early breastfeeding practices are associated with delivery location.

After adjusting for multiple confounders, higher odds of EIBF (39% higher) and no prelacteal feeding (75% higher) were seen amongst facility deliveries when compared to home deliveries. Marginally lower odds of no prelacteal feeding were observed in the private sector (11% lower) in comparison to the public sector, but no significant difference was seen between the sectors in terms of EIBF.

Higher odds of EIBF is seen in both facility deliveries and within the public sector – the consistency between both exposures highlights that EIBF is likely within control of delivery location as women remain there for the first hour post-partum. Not only do facility deliveries provide a point of contact with a health care professional who may provide advice about breastfeeding during the crucial time around birth [26], they could also mediate the effects of poorer, non-evidence based breastfeeding practices/advice received from within communities [27].

Potential reasons for lower odds of no prelacteal feeding within the private sector could include separation of the mother-infant dyad in the immediate post-partum period. This could be due to the increased availability of facilities, nurseries and health care professionals. Increased medical birth interventions within the private sector can also contribute to this separation [28]. Prelacteal feeding may then increase, specifically with formula, due to time spent away from the mother. Public sector facilities generally receive measured regulation and monitoring and may be more likely to implement evidence-based guidelines around breastfeeding, and adhere to WHO guidelines for quality of care [29] - this was seen in essential newborn care practices [30]. The *Baby Friendly Hospital Initiative (BFHI)* is an example of an initiative including informative courses, self-appraisal tools and external assessment tools that aim to encourage practices that protect, promote and support breastfeeding [31]. The outcome of no prelacteal feeding is more likely dependent on advice given within facilities (public or private) [32,33], rather than the facility themselves, as women tend to have left the facility by three days post-partum.

These findings highlight the window of opportunity that exists in the intrapartum and immediate postpartum period in order to positively impact early breastfeeding practices.

### Strengths and limitations

Strengths of this study include a large sample size of over 100 000 births, across 30 countries in SSA. Data was only included if births occurred within 24-month of the survey, following evidence that suggested short-term recall of breastfeeding is reliable, especially within a three-year period [34]. WHO IYCF indicators are also only in place for children 0-23 months old [35].

DHS data are based on respondents self-reporting, which relies upon the respondents’ knowledge of providers and accurate recall. Challenges encountered when relying on this self-reporting process for data collection include; conflation of responses, the varying terminology used between countries to describe different health care providers and professionals with different levels of experience and expertise. These cross-country variations were considered for this analysis following research which categorized delivery location and sector [36].

In this study, the categorization of delivery sector did not consider the likely differences within each sector. Across SSA the quality and provision of care around the time of birth varies greatly between tertiary and lower level facilities, despite being grouped together as response options on DHS survey data - for example, “private hospital/clinic”. Women included in the survey may also find it difficult to differentiate between sector providers when self-reporting for the survey. Private sector provision is also likely to differ by country, alongside inconsistencies in user fees, which are likely to impact the choice of delivery location. The concept of private and public sectors vary widely between countries. A better assessment of the different sector providers may be achieved if response options allowed for further divided into private for profit, faith based organisations (FBOs) and non-governmental organizations (NGOs) [28].

Home births included in this study could also be further stratified into skilled birth attendant (SBA) and non-SBA, which would allow for a better differentiation of the effect a health care professional has on favourable early breastfeeding outcomes, as was detailed in the descriptive analysis [22]. SBAs vary both in their level of training and role in achieving favourable early breastfeeding outcomes. However, we chose not to further differentiate home births as only a very small proportion of births were attended by SBA in our sample (5.2%) and SBA qualification from women’s self report has been shown to have low validity [37,38].

It is also worth noting this analysis has provided generalized results from a combination of 30 different countries, grouped together due to their geographical location. Country-specific results have shown some variability in the prevalence of the exposures and outcomes measured in this study, as well as the direction and significance of the four associations tested. Further research should allow for more detailed country-specific data in relation to favorable breastfeeding outcomes.

## CONCLUSION

The results of this study suggest that within SSA, the prevalence of EIBF and no prelacteal feeding is suboptimal, favourable early breastfeeding practices are more common amongst facility deliveries, and within facility deliveries they are more common amongst public sectors. These results suggest that care provided in the intrapartum and immediate post-partum period is crucial to increase the prevalence of favourable early breastfeeding practices. However, interpretation and recommendations following this study need to be reinforced through country and context specific understanding. Further research is required to ascertain which characteristics of care that may explain these observed trends, likely with country-specific data, improved data collection and better categorization of delivery sector.

## Additional material

Online Supplementary Document
