# Supplementary material for: Is childbirth location associated with higher rates of favourable early breastfeeding practices in Sub-Saharan Africa?
Source: J Glob Health. 2019 Feb 9;9(1):010417. doi: 10.7189/jogh.09.010417 (PMC6368939; doi:10.7189/jogh.09.010417)
Supplement: Online Supplementary Document [file jogh-09-010417-s001.pdf]

**Bergamaschi et al. J Glob Health 2019; 9: 010417**
**Online Supplementary Document**

**Table S1** -Characteristics of 103,611 women included in analysis, by country

| Country | Year of survey | Delivery location (%) | | Delivery Sector as a percentage of facility births* (%) | Early initiation of BF (%) | No prelacteal feeding (%) |
|---|---|---|---|---|---|---|
| | | **n** | **Facility** | **Public** | **Yes** | **Yes** |
| **Total** | | **103,611** | **46.91** | **77.82** | **50.02** | **61.01** |
| **Benin** | 2006 | 6,251 | 80.97 | 82.52 | 54.67 | 75.40 |
| **Burkina Faso** | 2010 | 5,727 | 73.94 | 98.37 | 42.35 | 63.32 |
| **Burundi** | 2010 | 2,832 | 69.90 | 92.83 | 74.76 | 92.35 |
| **Cameroon** | 2011 | 4,566 | 63.07 | 63.73 | 40.45 | 46.53 |
| **Chad** | 2004 | 2,041 | 14.11 | 86.37 | 32.62 | 2.71 |
| **Congo-B** | 2005 | 1,906 | 82.60 | 89.28 | 34.84 | 62.27 |
| **DRC** | 2007 | 3,377 | 72.40 | 70.85 | 48.63 | 83.03 |
| **Ethiopia** | 2011 | 4,115 | 11.48 | 87.34 | 52.50 | 72.00 |
| **Gabon** | 2012 | 2,434 | 92.92 | 72.00 | 33.59 | 52.28 |
| **Ghana** | 2008 | 1,187 | 58.88 | 83.61 | 52.66 | 82.10 |
| **Guinea** | 2005 | 2,490 | 29.26 | 95.56 | 38.29 | 27.00 |
| **Kenya** | 2008-9 | 2,316 | 45.53 | 75.29 | 56.90 | 57.09 |
| **Lesotho** | 2009 | 1,614 | 62.13 | 78.70 | 51.77 | 68.23 |
| **Liberia** | 2007 | 2,109 | 40.30 | 73.24 | 66.84 | 74.45 |
| **Madagascar** | 2008-9 | 4,587 | 34.97 | 91.63 | 71.44 | 73.96 |
| **Malawi** | 2010 | 7,341 | 79.88 | 77.82 | 95.56 | 96.79 |
| **Mali** | 2006 | 5,386 | 48.76 | 94.43 | 44.82 | 52.26 |
| **Mozambique** | 2011 | 4,285 | 60.28 | 95.95 | 77.64 | 92.94 |
| **Namibia** | 2006-7 | 2,076 | 81.67 | 94.90 | 68.06 | 80.64 |
| **Niger** | 2006 | 3,513 | 18.68 | 98.11 | 46.75 | 19.51 |
| **Nigeria** | 2008 | 10,623 | 36.31 | 57.43 | 37.38 | 42.08 |
| **Rwanda** | 2010 | 3,038 | 82.02 | 98.80 | 72.54 | 85.66 |
| **Sao Tome and Principe** | 2008-9 | 764 | 80.12 | 99.68 | 43.92 | 86.45 |
| **Senegal** | 2010-11 | 4,763 | 73.17 | 94.64 | 48.80 | 46.03 |
| **Sierra Leona** | 2008 | 2,297 | 24.84 | 88.46 | 49.42 | 38.50 |
| **Swaziland** | 2006-7 | 1,124 | 75.84 | 59.13 | 59.14 | 75.75 |
| **Tanzania** | 2010 | 3,056 | 51.83 | 81.63 | 46.07 | 67.88 |
| **Uganda** | 2011 | 2,919 | 60.05 | 76.67 | 53.36 | 58.05 |
| **Zambia** | 2007 | 2,531 | 47.35 | 89.82 | 56.48 | 90.13 |
| **Zimbabwe** | 2010-11 | 2,343 | 64.84 | 86.75 | 66.93 | 85.42 |

*denominator for this column is facility births only

**Figure S1. Conceptual framework for development of multivariable models**

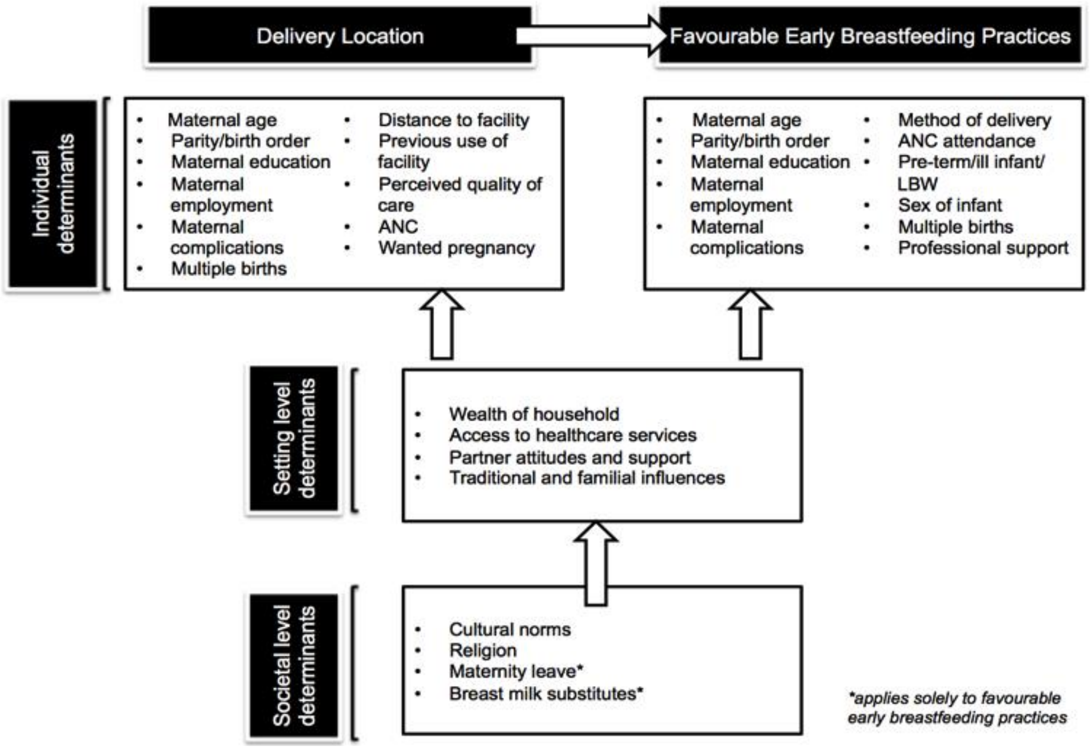

# Table S2. Full adjusted pooled models (continued from Table 3)

| | | Model 1 | | | Model 2 | | | Model 3 | | | Model 4 | | |
|---|---|---|---|---|---|---|---|---|---|---|---|---|---|
| | | Facility delivery and initiation of BF within 1 hour | | | Facility delivery and no prelacteal feeding | | | Private sector delivery initiation of BF within 1 hour | | | Private sector delivery and no prelacteal feeding | | |
| | | OR | 95% CI | P-value | OR | 95% CI | P-value | OR | 95% CI | P-value | OR | 95% CI | P-value |
| Maternal age | 15-19 | 0.91 | 0.82 – 1.01 | 0.020 | 0.79 | 0.70 – 0.88 | <0.001 | 0.96 | 0.84 – 1.10 | 0.696 | 0.81 | 0.70 – 0.94 | <0.001 |
| | 20- 24 | 1.02 | 0.96 – 1.10 | | 0.92 | 0.85 – 0.99 | | 1.04 | 0.94 – 1.15 | | 0.95 | 0.85 – 1.04 | |
| | 25-29 | 1 | | | 1.00 | | | 1.00 | | | 1.00 | | |
| | 30-34 | 1.08 | 0.99 – 1.16 | | 1.09 | 1.00 – 1.18 | | 0.99 | 0.89 – 1.09 | | 1.03 | 0.92 – 1.15 | |
| | 35-39 | 1.06 | 0.96 – 1.16 | | 0.99 | 0.91- 1.09 | | 0.93 | 0.82 – 1.06 | | 0.93 | 0.82 – 1.07 | |
| | 40-44 | 0.97 | 0.86 – 1.11 | | 1.04 | 0.90 – 1.20 | | 0.89 | 0.74 – 1.07 | | 0.84 | 0.67 – 1.06 | |
| | 45-49 | 1.18 | 0.98 – 1.42 | | 0.99 | 0.79 – 1.26 | | 0.84 | 0.61 – 1.16 | | 0.55 | 0.40 – 0.78 | |
| Parity | 1 | 0.76 | 0.71 – 0.82 | <0.001 | 0.84 | 1.78 – 0.91 | <0.01 | 0.74 | 0.67 – 0.81 | <0.001 | 0.79 | 0.71 – 0.88 | <0.001 |
| | 2-3 | 1 | | | 1.00 | | | 1.00 | | | 1.00 | | |
| | 4-5 | 1.03 | 0.97 – 1.10 | | 0.97 | 0.90 – 1.04 | | 1.10 | 1.00 – 1.21 | | 1.02 | 0.91 – 1.13 | |
| | 6+ | 0.95 | 0.87 – 1.04 | | 0.78 | 0.79 – 1.26 | | 1.01 | 0.89 – 1.15 | | 0.75 | 0.65 – 0.87 | |
| Maternal education | No education | 1 | | 0.038 | 1.00 | | <0.001 | 1.00 | | 0.167 | 1.00 | | <0.001 |
| | Primary | 1.13 | 1.05 – 1.22 | | 1.37 | 1.26 – 1.49 | | 1.11 | 0.99 – 1.24 | | 1.42 | 1.25 – 1.62 | |
| | Secondary and higher | 1.16 | 1.06 – 1.27 | | 1.30 | 1.18 – 1.45 | | 1.16 | 1.02 – 1.31 | | 1.24 | 1.06 – 1.45 | |
| Method of delivery | Vaginal | 1 | | <0.001 | 1.00 | | <0.001 | 1.00 | | <0.001 | 1.00 | | <0.001 |
| | C-section | 0.33 | 0.29 – 0.39 | | 0.53 | 0.46-0.61 | | 0.34 | 0.29 – 0.39 | | 0.56 | 0.49 – 0.65 | |
| ANC | No ANC | 1 | | 0.076 | 1.00 | | <0.001 | 1.00 | | 0.316 | 1.00 | | <0.001 |
| | 1-3 visits | 1.04 | 0.96 – 1.13 | | 1.27 | 1.15 – 1.39 | | 1.05 | 0.82 – 1.34 | | 1.34 | 1.07 – 1.68 | |
| | 4+ visits | 1.05 | 0.96 – 1.14 | | 1.44 | 1.31 – 1.58 | | 1.02 | 0.79 –1. 31 | | 1.51 | 1.21 – 1.88 | |
| Sex of infant | Male | 1 | | 0.010 | 1.00 | | <0.001 | 1.00 | | 0.068 | 1.00 | | 0.021 |
| | Female | 1.06 | 1.01 – 1.11 | | 1.09 | 1.04 – 1.15 | | 1.06 | 0.99 – 1.14 | | 1.08 | 1.01 – 1.17 | |
| Multiple births | Yes | 0.75 | 0.63 – 0.90 | 0.002 | 0.75 | 0.62 – 0.91 | 0.004 | 0.72 | 0.57 – 0.91 | 0.006 | 0.70 | 0.56 – 0.87 | 0.003 |
| | No | 1 | | | 1.00 | | | 1.00 | | | 1.00 | | |
| Wantedness of child | Wanted then | 1 | | <0.001 | 1.00 | | <0.001 | 1.00 | | <0.001 | 1.00 | | <0.001 |
| | Wanted later | 0.78 | 0.72 – 0.83 | | 0.87 | 0.81 – 0.93 | | 0.75 | 0.67 – 0.82 | | 0.82 | 0.75 – 0.90 | |
| | Wanted no more | 0.89 | 0.81 – 1.00 | | 1.12 | 1.01 – 1.27 | | 0.88 | 0.76 – 1.02 | | 1.09 | 0.94 – 1.27 | |
| Wealth quintile | Poorest | 1 | | <0.001 | 1.00 | | <0.001 | 1.00 | | 0.038 | 1.00 | | 0.007 |
| | Poorer | 1.07 | 0.99 – 1.15 | | 1.14 | 1.05 – 1.23 | | 1.05 | 0.94 – 1.17 | | 1.02 | 0.89 – 1.17 | |
| | Middle | 1.19 | 1.10 – 1.29 | | 1.22 | 1.11 – 1.34 | | 1.13 | 0.99 – 1.28 | | 0.93 | 0.81 – 1.06 | |
| | Richer | 1.17 | 1.06 – 1.29 | | 1.23 | 1.10 – 1.38 | | 1.12 | 0.97 – 1.28 | | 0.89 | 0.76 – 1.05 | |
| | Richest | 1.19 | 1.06 – 1.34 | | 1.14 | 0.98 – 1.32 | | 1.12 | 0.97 – 1.30 | | 0.87 | 0.72 – 1.04 | |
| Residence | Urban | 0.99 | 0.91 – 1.10 | 0.952 | 0.90 | 0.80 – 1.00 | 0.058 | 1.07 | 0.96 – 1.19 | 0.247 | 0.96 | 0.85 – 1.10 | 0.601 |
| | Rural | 1 | | | 1.00 | | | 1.00 | | | 1.00 | | |
| Marital status | Ever married | 1 | | 0.584 | 1.00 | | 0.765 | 1.00 | | 0.998 | 1.00 | | 0.959 |
| | Never married | 1.04 | 0.91 – 1.19 | | 0.98 | 0.85 – 1.12 | | 1.00 | 0.85 – 1.17 | | 1.00 | 0.83 – 1.20 | |
| Country | | see SM4 for country-level effect estimates | | | see SM4 for country-level effect estimates | | | see SM4 for country-level effect estimates | | | see SM4 for country-level effect estimates | | |

*P-values of likelihood ratio test*

## Table S3. Country-level effect estimates from adjusted models in Table 3

| | Model 1 | | | Model 2 | | |
|---|---|---|---|---|---|---|
| **Exposure** | Facility delivery* | | | Facility Delivery* | | |
| **Outcome** | Early breastfeeding initiation | | | No prelacteal feeding | | |
| **Country** | **aOR** | **95%CI** | **P-value** | **aOR** | **95%CI** | **P-value** |
| **Benin** | 2.52 | 2.03-3.13 | <0.001 | 2.76 | 2.21 - 3.45 | <0.001 |
| **Burkina Faso** | 1.48 | 1.23 - 1.79 | <0.001 | 2.79 | 2.27 - 3.43 | <0.001 |
| **Burundi** | 1.22 | 0.97 - 1.54 | 0.086 | 1.42 | 0.89 - 2.25 | 0.134 |
| **Cameroon** | 1.24 | 1.01 - 1.51 | 0.042 | 2.06 | 1.62 - 2.60 | <0.001 |
| **Chad** | 1.15 | 0.84 - 1.57 | 0.382 | 1.58 | 0.84 - 2.97 | 0.151 |
| **Congo-B** | 0.92 | 0.65 - 1.31 | 0.656 | 1.48 | 1.05 - 2.10 | 0.026 |
| **DRC** | 1.06 | 0.75 - 1.50 | 0.736 | 0.95 | 0.59 - 1.54 | 0.839 |
| **Ethiopia** | 0.89 | 0.57 - 1.36 | 0.585 | 1.36 | 0.91 - 2.04 | 0.132 |
| **Gabon** | 0.45 | 0.27 - 0.72 | <0.001 | 0.92 | 0.59 - 1.43 | 0.703 |
| **Ghana** | 1.45 | 1.05 - 2.00 | 0.024 | 1.34 | 0.82 - 2.19 | 0.248 |
| **Guinea** | 1.10 | 0.86 - 1.41 | 0.422 | 1.77 | 1.34 - 2.35 | <0.001 |
| **Kenya** | 1.47 | 1.18 - 1.95 | 0.006 | 2.69 | 1.92 - 3.76 | <0.001 |
| **Lesotho** | 1.26 | 0.99 - 1.62 | 0.060 | 2.51 | 1.85 - 3.40 | <0.001 |
| **Liberia** | 0.67 | 0.49 - 0.92 | 0.014 | 1.07 | 0.77 - 1.48 | 0.694 |
| **Madagascar** | 1.24 | 1.01 - 1.52 | 0.042 | 1.57 | 1.25 - 1.96 | <0.001 |
| **Malawi** | 1.12 | 0.77 - 1.64 | 0.550 | 2.02 | 1.35 - 3.01 | <0.001 |
| **Mali** | 1.35 | 1.09 - 1.68 | 0.006 | 1.91 | 1.56 - 2.33 | <0.001 |
| **Mozambique** | 1.25 | 1.01 - 1.56 | 0.045 | 1.34 | 0.89 - 2.01 | 0.155 |
| **Namibia** | 1.64 | 1.16 - 2.32 | 0.005 | 2.06 | 1.44 - 2.05 | <0.001 |
| **Niger** | 1.63 | 1.24 - 2.14 | <0.001 | 1.29 | 0.90 - 1.84 | 0.171 |
| **Nigeria** | 1.24 | 1.06 - 1.45 | 0.006 | 1.85 | 1.59 - 2.14 | <0.001 |
| **Rwanda** | 2.01 | 1.59 - 2.53 | <0.001 | 2.67 | 2.03 - 3.53 | <0.001 |
| **Sao Tome and Principe** | 1.10 | 1.06 - 1.45 | 0.006 | 0.98 | 0.47 - 2.02 | 0.947 |
| **Senegal** | 1.39 | 1.11 - 1.74 | 0.004 | 1.23 | 1.00 - 1.52 | 0.049 |
| **Sierra Leona** | 1.00 | 0.75 - 1.33 | 0.991 | 1.49 | 1.09 - 2.04 | 0.012 |
| **Swaziland** | 1.92 | 1.36 - 2.70 | <0.001 | 1.94 | 1.32 - 2.87 | <0.001 |
| **Tanzania** | 2.66 | 2.15 - 3.28 | <0.001 | 2.1 | 1.65 - 2.69 | <0.001 |
| **Uganda** | 1.26 | 1.02 - 1.55 | 0.029 | 1.44 | 1.16 - 1.79 | <0.001 |
| **Zambia** | 1.6 | 1.27 - 2.01 | <0.001 | 1.38 | 0.99 - 1.92 | 0.056 |
| **Zimbabwe** | 2.24 | 1.75 - 2.86 | <0.001 | 2.08 | 1.55 - 2.78 | <0.001 |

*Comparing to home delivery. ** comparing to public sector facility delivery (sub-set of facility deliveries only)
Adjusted for maternal age group, parity, maternal education, method of delivery, number of ANC visits, sex of infant, multiple births, wantedness of child, wealth quintile, residence, and marital category.

| | **Model 3** | | | **Model 4** | | |
|---|---|---|---|---|---|---|
| **Exposure** | Private delivery** | | | Private delivery** | | |
| **Outcome** | Early breastfeeding initiation | | | No prelacteal feeding | | |
| **Country** | **aOR** | **95%CI** | **P-value** | **aOR** | **95%CI** | **P-value** |
| **Benin** | 0.87 | 0.71 - 1.08 | 0.208 | 0.69 | 0.57 - 0.84 | <0.001 |
| **Burkina Faso** | 2.58 | 1.20 - 5.54 | 0.015 | 0.97 | 0.43 - 2.19 | 0.940 |
| **Burundi** | 0.85 | 1.20 - 5.54 | 0.015 | 1.004 | 0.51 - 1.98 | 0.990 |
| **Cameroon** | 1.02 | 0.85 - 1.23 | 0.839 | 1.43 | 1.16 - 1.75 | <0.001 |
| **Chad** | 1.07 | 0.45 - 2.54 | 0.88 | 3.07 | 0.81 -11.61 | 0.098 |
| **Congo-B** | 1.52 | 1.02 - 2.27 | 0.041 | 1.45 | 0.94 - 2.25 | 0.095 |
| **DRC** | 0.97 | 0.74 - 1.27 | 0.831 | 0.80 | 0.60 - 1.06 | 0.114 |
| **Ethiopia** | 0.59 | 2.38 - 1.47 | 0.258 | 0.89 | 0.36 - 2.24 | 0.805 |
| **Gabon** | 1.19 | 0.86 - 1.63 | 0.288 | 1.02 | 0.72 - 1.45 | 0.925 |
| **Ghana** | 0.76 | 0.46 - 1.25 | 0.279 | 1.05 | 0.59 - 2.05 | 0.753 |
| **Guinea** | 1.60 | 0.57 - 4.55 | 0.372 | 1.16 | 0.45 - 3.03 | 0.761 |
| **Kenya** | 1.42 | 0.94 - 2.14 | 0.093 | 1.08 | 0.71 - 1.64 | 0.727 |
| **Lesotho** | 0.82 | 0.55 - 1.24 | 0.345 | 0.69 | 0.45 - 1.04 | 0.075 |
| **Liberia** | 0.9 | 0.54 - 1.50 | 0.694 | 1.82 | 0.97 - 3.38 | 0.060 |
| **Madagascar** | 0.69 | 0.39 - 1.24 | 0.216 | 0.43 | 0.26 - 0.72 | <0.001 |
| **Malawi** | 1.83 | 1.14 - 2.94 | 0.013 | 0.62 | 0.39 - 0.97 | 0.036 |
| **Mali** | 1.14 | 0.65 - 1.99 | 0.643 | 1.04 | 0.58 - 1.89 | 0.889 |
| **Mozambique** | 1.92 | 0.99 - 3.71 | 0.051 | 1.74 | 0.61 - 4.99 | 0.302 |
| **Namibia** | 0.81 | 0.40 - 1.67 | 0.573 | 0.82 | 0.41 - 1.63 | 0.562 |
| **Niger** | 0.31 | 1.14 - 0.71 | 0.006 | 0.80 | 0.34 - 1.88 | 0.612 |
| **Nigeria** | 0.69 | 0.57 - 0.84 | <0.001 | 0.83 | 0.69 - 0.99 | 0.045 |
| **Rwanda** | 0.71 | 0.31 - 1.60 | 0.407 | 0.18 | 0.09 - 0.35 | <0.001 |
| **Sao Tome and Principe** | Estimates not available due to insufficient sample of deliveries in private sector | | | | | |
| **Senegal** | 0.85 | 0.51 - 1.41 | 0.521 | 0.63 | 0.35 - 1.11 | 0.109 |
| **Sierra Leona** | 0.40 | 0.15 - 1.04 | 0.061 | 0.58 | 0.25 - 1.36 | 0.208 |
| **Swaziland** | 0.80 | 0.58 - 1.11 | 0.178 | 0.84 | 0.59 - 1.18 | 0.304 |
| **Tanzania** | 1.24 | 0.83 - 1.86 | 0.296 | 0.96 | 0.62 - 1.46 | 0.834 |
| **Uganda** | 1.29 | 0.97 - 1.72 | 0.075 | 0.96 | 0.70 - 1.33 | 0.824 |
| **Zambia** | 0.67 | 0.42 - 1.07 | 0.096 | 0.71 | 0.36 - 1.38 | 0.312 |
| **Zimbabwe** | 0.78 | 0.54 - 1.15 | 0.208 | 0.72 | 0.43 - 1.23 | 0.229 |

*Comparing to home delivery. ** comparing to public sector facility delivery (sub-set of facility deliveries only)
Adjusted for maternal age group, parity, maternal education, method of delivery, number of ANC visits, sex of infant, multiple births, wantedness of child, wealth quintile, residence, and marital category.
